# Silencing SAPCD2 Represses Proliferation and Lung Metastasis of Fibrosarcoma by Activating Hippo Signaling Pathway

**DOI:** 10.3389/fonc.2020.574383

**Published:** 2020-12-15

**Authors:** Bowen Zhu, Yanqin Wu, Lizhi Niu, Wang Yao, Miao Xue, Hongyu Wang, Jianyong Yang, Jiaping Li, Wenzhe Fan

**Affiliations:** ^1^ Department of Interventional Oncology, The First Affiliated Hospital of Sun Yat-Sen University, Guangzhou, China; ^2^ Fuda Cancer Hospital, Jinan University School of Medicine, Guangzhou, China; ^3^ Fudan Institute of Cryosurgery for Cancer, Jinan University School of Medicine, Guangzhou, China; ^4^ Department of Interventional Radiology, The First Affiliated Hospital of Sun Yat-Sen University, Guangzhou, China; ^5^ Department of Medical Imaging, The First Affiliated Hospital of Sun Yat-Sen University, Guangzhou, China

**Keywords:** SAPCD2, proliferation, lung metastasis, Hippo signaling, fibrosarcoma

## Abstract

The primary problem associated with fibrosarcoma is its high potential to metastasize to the lung. Aberrant expression of SAPCD2 has been widely reported to be implicated in the progression and metastasis in multiple cancer types. However, the clinical significance and biological roles of SAPCD2 in fibrosarcoma remain unknown. Here, we reported that SAPCD2 expression was markedly elevated in fibrosarcoma tissues, and its expression was differentially upregulated in fibrosarcoma cell lines compared with that in several primary fibroblast cell lines. Kaplan-Meier survival analysis revealed that SAPCD2 overexpression was significantly correlated with early progression and metastasis, and poor prognosis in fibrosarcoma patients. Our results further showed that silencing SAPCD2 inhibited the proliferation and increased the apoptosis of fibrosarcoma cells *in vitro*. Importantly, silencing SAPCD2 repressed lung metastasis of fibrosarcoma cells *in vivo*. Mechanistic investigation further demonstrated that silencing SAPCD2 inhibited the proliferation and lung metastasis of fibrosarcoma cells by activating the Hippo signaling pathway, as evidenced by the finding that constitutively active YAP1, YAP1-S127A, significantly reversed the inhibitory effect of SAPCD2 downregulation on the colony formation and anchorage-independent growth capabilities of fibrosarcoma cells, as well as the stimulatory effect on the apoptotic ratio of fibrosarcoma cells. In conclusion, SAPCD2 promotes the proliferation and lung metastasis of fibrosarcoma cells by regulating the activity of Hippo signaling, and this mechanism represents a potential therapeutic target for the treatment of lung metastatic fibrosarcoma.

## Introduction

Fibrosarcoma is a rare, highly malignant tumor of mesenchymal cell origin, and it is thought to be the most common malignant soft-tissue sarcoma in adults (1), accounting for approximately 3.6% of all malignant sarcomas in adults with a peak age ranging from 30 to 60 years old (2). Pathologically, fibrosarcoma is mainly derived from transformed spindle shaped fibroblasts characterized by an excessively high rate of division. The response rate of fibrosarcoma to radiation and chemotherapy has been reported to be low, although these treatments are broadly used as neoadjuvant and/or adjuvant therapeutic strategies (3). In this context, identification of novel gene candidates implicated in the development and progression of fibrosarcoma may facilitate accurate diagnosis and appropriate treatment plans.

Recently, the metastatic behavior of cancer, which contributes to the vast majority of cancer-related deaths (4), has increasing been a primary area of interest. Tumor metastasis is a progressive and dynamic process, which begins with escape from the primary tumor through epithelial-mesenchymal transition (EMT), continues to degradation of the extracellular matrix, intravasation into the blood/lymph vessel system, survival through anoikis resistance, extravasation from the blood/lymph vessel system, and colonization of and metastasis to distant organs, and ultimately results in the of new tumors in secondary organs through cell proliferation (5, 6). Fibrosarcoma is a type of cancer with high metastatic potential, and the most frequently observed metastatic sites of fibrosarcoma include the lung and lymph nodes. Among these metastasis, pulmonary metastases are a well-known complication of fibrosarcomatous tumors, which is common for these lesions to occur bilaterally (7–10). After metastasis to the lung, the formation of metastatic pulmonary tumors results in significant morbidity and poor survival due to late detection and diagnosis of fibrosarcoma at an advanced stage. Therefore, further identification of the lung metastasis-associated markers is of great importance for the early detection of lung metastasis and the development of novel anti-lung metastasis therapeutic strategies for the treatment of fibrosarcoma.

Since it was first identified in gastric cancer (11), suppressor anaphase-promoting complex domain containing 2 (SAPCD2), also referred to as C9orf140 or p42.3, has been shown to play an important role in controlling spindle orientation and divisions as a cell cycle-dependent factor (12). SAPCD2 located on human chromosome 9q42.3 encodes a 389-amino acid protein (42.3 kDa) with EF-Hand domain at the N-terminal and functional CC-domains at the C-terminus (13). Accumulating studies have reported that SAPCD2 is overexpressed in several types of human cancer, and that SAPCD2 overexpression promotes the proliferation, migration, and invasion of cancer cells by various mechanisms (11, 14–20). However, little is known about the clinical significance and functional role of SAPCD2 in fibrosarcoma, especially metastatic fibrosarcoma.

In the current study, our results demonstrated that SAPCD2 was upregulated in fibrosarcoma tissues and cell lines, and SAPCD2 overexpression predicted early progression and metastasis, and poor prognosis in fibrosarcoma patients. Loss-of-function experiments showed that silencing SAPCD2 inhibited the proliferation and increased the apoptosis of fibrosarcoma cells *in vitro*, and repressed the lung metastasis of fibrosarcoma cells *in vivo*. Our results further revealed that silencing SAPCD2 inhibited the proliferation and lung metastasis of fibrosarcoma cells by activating the Hippo signaling pathway. Therefore, our results reveal a novel mechanism by which SAPCD2 promotes the proliferation and lung metastasis of fibrosarcoma cells.

## Materials and Methods

### Cell Lines and Cell Culture

The normal fibroblast cells human dermal fibroblast primary cell (hDFPC), human lung fibroblasts primary cell (hLFPC), human mammary fibroblasts primary cell (hMFPC), human embryonic lung fibroblast (HFL1), and one fibrosarcoma cell HT-1080 were obtained from Procell (Wuhan, China). Another fibrosarcoma cell SW684 was obtained from American Type Culture Collection (ATCC, Manassas, VA, USA). All cells cultured at 37°C in a humidified atmosphere with 5% CO_2_, and in different complete medium. hDFPC(CM-H103), hLFPC(CM-H011), hMFPC(CM-H172), HFL1(CM-H159), HT-1080(CM-0117), and SW684 was routinely cultured in Dulbecco’s Modified Eagle Medium (DMEM) medium supplemented with 10% fetal bovine serum (FBS), 2 mM L-glutamine and penicillin/streptomycin (i.e., complete medium).

### Patients and Tumor Tissues

All individual paraffin-embedded, archived samples, 54 benign fibroma tissues (**Table 1**), 48 dermatofibrosarcoma protuberans tissues without fibrosarcomatous change (**Table 2**), and 59 fibrosarcoma tissues (**Table 3**), were obtained during surgery at The First Affiliated Hospital of Sun Yat-sen University (Guangzhou, China) and Fuda Cancer Hospital, Jinan University School of Medicine (Guangzhou, China) between Between January 2008 and December 2013. Patients were diagnosed based on clinical and pathological evidence, and the specimens were immediately snap-frozen and stored in liquid nitrogen tanks. All sample tissues used in the current study were determined by two independent experienced pathologists. For the use of these clinical materials for research purposes, prior patients’ consents and approval from the Institutional Research Ethics Committee were obtained.

**Table 1:** The basic information of 54 patients with fibroma for SAPCD2 immunohistochemical staining analysis.

		Cases (n)	Percentage (%)
Gender	Female	34	63.0
	Male	20	37.0
Age	<60	43	79.6
	≥60	11	20.4
Pathological type	Fibroma	54	100.0
	Other	0	0.0
Location	Breast	5	9.3
	Ovary	7	13.0
	Other skin	33	61.1
	Other deep soft tissue	9	16.7

**Table 2:** The basic information of 48 patients with dermatofibrosarcoma protuberans for SAPCD2 immunohistochemical staining analysis.

		Cases (n)	Percentage (%)
Gender	Female	16	33.3
	Male	32	66.7
Age	<60	34	70.8
	≥60	14	29.2
Pathological type	Dermatofibrosarcoma protuberans	48	100.0
	Other	0	0.0
Location	Body	23	47.9
	Arms and legs	18	37.5
	Head	7	14.6

**Table 3:** The basic information of 59 patients with fibrosarcoma for SAPCD2 immunohistochemical staining analysis.

		Cases (n)	Percentage (%)
Gender	Female	25	42.4
	Male	34	57.6
Age	<60	44	74.6
	≥60	15	25.4
Pathological type	Fibrosarcoma	59	100.0
	Other	0	0.0
Location	Body	14	23.7
	Arms and legs	14	23.7
	Head	12	20.3
	Deep soft tissue	19	32.2
Margin status	Positive	17	28.8
	Negative	42	71.2
Metastatic status	Positive	12	20.3
	Negative	47	79.7

### RNA Extraction, Reverse Transcription, and Real-Time PCR

Total RNA from tissues or cells was extracted using TRIzol (Life Technologies) according to the manufacturer’s instructions. Messenger RNA (mRNA) were polyadenylated using a poly-A polymerase-based First-Strand Synthesis kit (TaKaRa, DaLian, China) and reverse transcription (RT) of total mRNA was performed using a PrimeScript RT Reagent kit (TaKaRa) according to the manufacturer’s protocol. Complementary DNA (cDNA) was amplified and quantified on ABI 7500HT system (Applied Biosystems, Foster City, CA, USA) using SYBR Green I (Applied Biosystems). The primers used in the reactions were listed in **Table 4**. Real-time PCR was performed as described previously (21). Glyceraldehyde-3-phosphate dehydrogenase (GAPDH) was used as endogenous controls. Relative fold expressions were calculated with the comparative threshold cycle (2^−ΔΔCt^) method according to the previous study (22).

**Table 4:** A list of primers used in the reactions for real-time RT-PCR.

Gene	Sequence (5′–3′)
SAPCD2-up	TGCCCAAGGTACAAGAGGTG
SAPCD2-dn	CTGGGTGAGGAGTCGGTTCT
GAPDH-up	TCCTCTGACTTCAACAGCGACAC
GAPDH-dn	CACCCTGTTGCTGTAGCCAAATTC
CTGF-up	TGGAGATTTTGGGAGTACGG
CTGF-dn	CAGGCTAGAGAAGCAGAGCC
CYR61-up	GGTCAAAGTTACCGGGCAGT
CYR61-dn	GGAGGCATCGAATCCCAGC
HOXA1-up	TCCTGGAATACCCCATACTTAGC
HOXA1-dn	GCACGACTGGAAAGTTGTAATCC
SOX9-up	AGCGAACGCACATCAAGAC
SOX9-dn	CTGTAGGCGATCTGTTGGGG
RPL13A-up	GCCATCGTGGCTAAACAGGTA
RPL13A-dn	GTTGGTGTTCATCCGCTTGC
PPIA-up	GGCAAATGCTGGACCCAACACA
PPIA-dn	TGCTGGTCTTGCCATTCCTGGA

### Plasmid and Transfection

Knockdown of endogenous SAPCD2 was performed by cloning two short hairpin RNA (shRNA) oligonucleotides into the pSUPER-puro-retro vector (OligoEngine, Seattle, WA, USA). Two separate shRNA fragments of SAPCD2 are listed in **Table 5**. Transfection of plasmids was performed using Lipofectamine 3000 (Life Technologies) according to the manufacturer’s instructions.

**Table 5:** A list of primers used in the reactions for clone PCR.

Gene	Sequence (5′–3′)
shSAPCD2-1#-up	CCGGACCTCTGGATTCCACCTTCATCTCGAGATGAAGGTGGAATCCAGAGGTTTTTTTG
shSAPCD2-1#-dn	AATTCAAAAAACCTCTGGATTCCACCTTCATCTCGAGATGAAGGTGGAATCCAGAGGTT
shSAPCD2-2#-up	CCGGGCAGCAGCAGACCATCCTCATCTCGAGATGAGGATGGTCTGCTGCTGCTTTTTTG
shSAPCD2-2#-dn	AATTCAAAAAGCAGCAGCAGACCATCCTCATCTCGAGATGAGGATGGTCTGCTGCTGCT

### Western Blotting Analysis

Nuclear/cytoplasmic fractionation was separated by using Cell Fractionation Kit (Cell Signaling Technology, USA) according to the manufacturer’s instructions, and the whole cell lysates were extracted using RIPA Buffer (Cell Signaling Technology). Western blot was performed according to a standard method, as described previously (23). Antibodies against p-MST1/2, MST1, p-LATS1, LATS1, p-YAP1, and YAP1 were purchased from Cell Signaling Technology, TAZ from Abcam, and SAPCD2 from ThermoFisher. The membranes were stripped and reprobed with an anti–α-tubulin antibody (Cell Signaling Technology) as the loading control.

### Anchorage-Independent Growth Assay

A total of 500 cells were trypsinized and suspended in 2 ml of complete medium containing 0.3% agar (Sigma). This experiment was performed as previously described (24) and carried out three times independently for each cell line.

### Flow Cytometric Analysis

Flow cytometric analyzed of apoptosis were used the Annexin V-FITC/PI Apoptosis Detection Kit (KeyGEN, China), and was presented as protocol described (25). The cell’s inner mitochondrial membrane potential (Δψm) was detected by flow cytometric using MitoScreen JC-1 staining kit (BD) (26). Briefly, cells were dissociated with trypsin and resuspended at 1 × 10^6^ cells/ml in Assay Buffer, and then incubated at 37°C for 15 min with 10 μl/ml JC-1. Before analyzed by flow cytometer, cells were washed twice by Assay Buffer. Flow cytometry data were analyzed using FlowJo 7.6 software (TreeStar Inc., USA) as previously described (27).

### Cell Counting Kit-8 Analysis

For cell counting kit-8 analysis, cells (2 × 10^3^) were seeded into 96-well plates and the specific staining process and methods were performed according to the previous study (28).

### Colony Formation Assay

The cells were trypsinized as single cell and suspended in the media with 10% FBS. The indicated cells (300 cells per well) were seeded into of 6-well plate for ~10–14 days. Colonies were stained with 1% crystal violet for 10 min after fixation with 10% formaldehyde for 5 min. Plating efficiency was calculated as previously described (29). Different colony morphologies were captured under a light microscope (Olympus).

### Invasion Assay

The invasion assay was performed using Transwell chamber consisting of 8-mm membrane filter inserts (Corning) with coated Matrigel (BD Biosciences) as described previously (30). Briefly, the cells were trypsinized and suspended in serum-free medium. Then, 1.5 × 105 cells were added to the upper chamber, and lower chamber was filled with the culture medium supplemented with 10% FBS. After incubation for 24–48 h, cells passed through the coated membrane to the lower surface, where cells were fixed with 4% paraformaldehyde and stained with haematoxylin. The cell count was performed under a microscope (×100).

### Gene Set Enrichment Analysis

Gene set enrichment analysis (GSEA) was performed using GSEA 2.2.1 (http://www.broadinstitute.org/gsea) as previously described (24). Briefly, we first downloaded the mRNA sequencing dataset of Fibrosarcoma from TCGA and procure the expression value of the corresponding genes from the Level 3 data of each sample (The unit was RNA-Seq by Expectation Maximization, RSEM); analyze the log2 value of each sample using Excel 2010 and GraphPad 5, as well as statistically analyze the RNA expression level of all Fibrosarcoma tissues using paired t-test or unpaired t-test performed by SPSS version 19.0 (SPSS Inc., Chicago, IL, USA); GSEA was performed with RNA sequencing dataset of Fibrosarcoma from TCGA as Expression dataset. The high and low expression level of SAPCD2 was stratified by the medium expression level of SAPCD2 in all Fibrosarcoma tissues. Gene set was performed by Molecular Signatures Database v5.2 (http://software.broadinstitute.org/gsea/msigdb) (all processing parameters as the default).

### Animal Study

Four-week-old BALB/c-nu female mice weighing 15–20 g were maintained in a standard pathogen-free environment where the animals were housed in sterile cages under laminar flow hoods in a 20–26°C temperature controlled room with a 12-h light/12-h dark cycle and fed autoclaved chow and water. BALB/c-nu mice at 4–6 weeks of age were randomly divided into three groups (n = 6 per group) and the indicated HT-1080 cells (1 × 10^6^) were injected into the lateral tail vein of the nude mice. The mice were sacrificed by inhaling CO2 on 36 days after inoculation. Lungs of each group of mice were dissected and fixed with 4% paraformaldehyde. Limbs were decalcified by gentle shaking in TE decalcifying solution (pH 7.4) for 4 weeks. All the tissues were finally paraffin embedded and subjected to hematoxylin and eosin (H&E). The number of tumor cell per mm^2^ was calculated as the previous study (31).

### Immunohistochemistry

The immunohistochemistry procedure and scoring of SAPCD2 expression levels were performed as previously described (29). Scores given by two independent investigators were averaged for further comparative evaluation of SAPCD2 expression. The proportion of tumor cells was scored as follows: 0 (no positive tumor cells); 1 (<10% positive tumor cells); 2 (10–35% positive tumor cells); 3 (35–70% positive tumor cells); and 4 (>70% positive tumor cells). The staining intensity (SI) was graded according to the following criteria: 0 (no staining); 1 (weak staining, light yellow); 2 (moderate staining, yellow brown); and 3 (strong staining, brown). The staining index (SI) was calculated as the product of the SI score and the proportion of positive tumor cells. Using this method of assessment, we evaluated SAPCD2 expression in Fibrosarcoma samples by determining SI, with scores of 0, 1, 2, 3, 4, 6, 8, 9, or 12.

### Luciferase Assay

Cells (4 × 10^4^) were seeded in triplicate in 24-well plates and cultured for 24 h, and the luciferase reporter assay was performed as previously described (32). Cells were transfected with 100 ng HOP-Flash (Catalog # 83467, Addgene) or HIP-Flash luciferase reporter plasmid (Catalog # 83466, Addgene), plus 5 ng pRL-TK Renilla plasmid (Promega) using Lipofectamine 3000 (Invitrogen) according to the manufacturer’s recommendation. Luciferase and Renilla signals were measured 36 h after transfection using a Dual Luciferase Reporter Assay Kit (Promega) according to the manufacturer’s protocol.

### Statistical Analysis

All values are presented as means ± standard deviation (SD). Significant differences were determined using GraphPad 5.0 software (USA). Student’s t-test was used to determine statistical differences between two groups. One-way ANOVA was used to determine statistical differences between multiple testing. The chi-square test was used to analyze the relationship between SAPCD2 expression and clinicopathological characteristics. Survival curves were plotted using the Kaplan Meier method and compared by log-rank test. P < 0.05 was considered significant. All the experiments were repeated three times.

## Results

### SAPCD2 Expression Is Increased in Fibrosarcoma Tissues and Cell Lines

**Figure 1:**
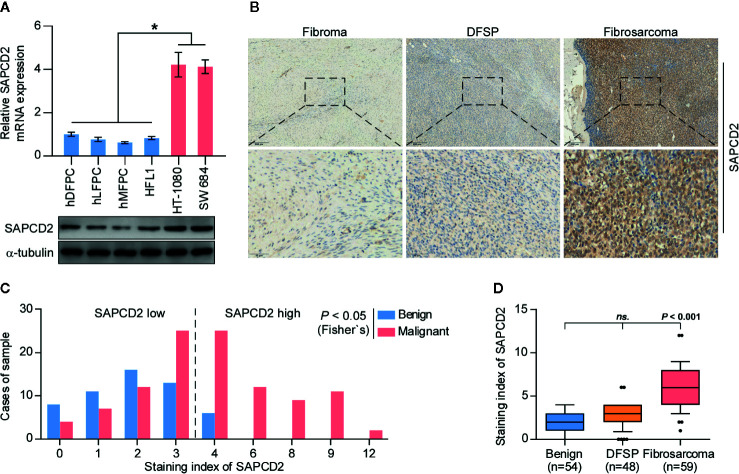
SAPCD2 is upregulated in fibrosarcoma tissues and cells. **(A)** Real-time PCR and Western blotting analysis of SAPCD2 expression in 4 normal fibroblast cell lines, including human dermal fibroblast primary cell (hDFPC), human lung fibroblasts primary cell (hLFPC), human mammary fibroblasts primary cell (hMFPC), Human embryonic lung fibroblast (HFL1), and 2 fibrosarcoma cell lines HT-1080 and SW684. GAPDH was used as endogenous controls in RT-PCR and α-Tubulin was detected as a loading control in the Western blot. Each bar represents the mean values ± SD of three independent experiments. *P < 0.05. **(B)** Representative images of SAPCD2 expression in benign fibroma, dermatofibrosarcoma protuberans (DFSP), and fibrosarcoma tissues. **(C)** The number of fibroma, DFSP, and fibrosarcoma tissues stratified by staining index of IHC. **(D)** Immunohistochemical staining index of SAPCD2 in fibroma, DFSP, and fibrosarcoma tissues. n.s. indicates no significance.

First, the expression of SAPCD2 in hDFPCs, human lung fibroblast primary cells (hLFPCs), human mammary fibroblast primary cells (hMFPCs), human embryonic lung fibroblasts (HFL1), and two fibrosarcoma cell lines, HT-1080 and SW 684, was examined by real-time PCR and western blot. As shown in **Figure 1A**, the mRNA and protein expression levels of SAPCD2 were dramatically upregulated in the HT-1080 cells and SW 684 cells compared with those in the four noncancerous fibroblast cell lines. We further measured SAPCD2 expression in 54 benign fibroma samples, 48 low-level malignant dermatofibrosarcoma protuberans without fibrosarcomatous change (DFSP) samples and 59 highly malignant fibrosarcoma samples by immunohistochemical staining (IHC), and found that SAPCD2 expression was significantly elevated in the fibrosarcoma tissues compared with that in the benign fibroma tissues and DFSP tissues (**Figures 1B–D**). These findings demonstrated that SAPCD2 is upregulated in clinical fibrosarcoma tissues and cell lines.

### Overexpression of SAPCD2 Is Correlated With Early Progression and Metastasis, and Poor Prognosis

**Figure 2:**
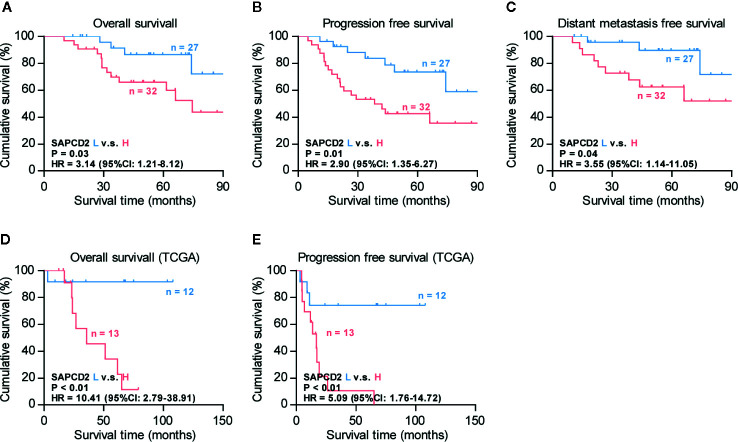
Overexpression of SAPCD2 correlates with advanced clinicopathological features and poor prognosis in fibrosarcoma patients. **(A**–**C)** Kaplan-Meier analysis of overall survival (OS) **(A)**, progression-free survivals (PFS) **(B)**, and distant metastasis-free survival (DMFS) **(C)** in fibrosarcoma patients with low SAPCD2 expression versus high SAPCD2 expression. **(D**–**E)** Kaplan-Meier analysis of overall survival (OS) **(D)** and progression-free survivals (PFS) **(E)** in fibrosarcoma patients with low SAPCD2 expression versus high SAPCD2 expression from TCGA.

The clinical significance of SAPCD2 in fibrosarcoma was further investigated. Based on the SI of the 66 fibrosarcoma samples examined above by IHC, low and high SAPCD2 expression was stratified by the following criteria: SI ≤ 3 was used to define fibrosarcoma tissues with low SAPCD2 expression, and SI ≥ 4 was used to define fibrosarcomatissues with high SAPCD2 expression. Kaplan-Meier survival analysis further showed that fibrosarcoma patients with high SAPCD2 expression had poorer overall survival than those with low SAPCD2 expression (**Figure 2A**). Furthermore, compared with downexpression of SAPCD2, overexpression of SAPCD2 predicted shortened progression-free and distant metastasis-free survival in fibrosarcoma patients (**Figures 2B, C**). Statistical analysis revealed that overexpression of SAPCD2 was significantly and positively correlated with metastatic status in fibrosarcoma patients (P = 0.028, **Table 6**). Consistently, our results were further supported by the findings from the fibrosarcoma datasets from TCGA that fibrosarcoma patients with high SAPCD2 expression exhibited poor overall survival and early progression-free survival compared with those with low SAPCD2 expression (**Figures 2D, E**). Therefore, our results indicated that overexpression of SAPCD2 is significantly correlated with early progression and metastasis, and poor overall prognosis in fibrosarcoma patients.

**Table 6:** The relationship between SAPCD2 protein level and clinical pathological characteristics in 59 patients with fibrosarcoma.

Parameters	Number of cases	SAPCD2 IHC-SI		P values
		Low	High	
Gender				
Female	25	11	14	0.816
Male	34	16	18	
Age				
<60	44	21	23	0.604
≥60	15	6	9	
Margin status				
Positive	17	6	11	0.305
Negative	42	21	21	
Metastatic status				
Positive	12	2	10	0.028*
Negative	47	25	22	

### Silencing SAPCD2 Inhibits Lung Colonization and Primary Growth of Fibrosarcoma Cells *In Vivo*

**Figure 3:**
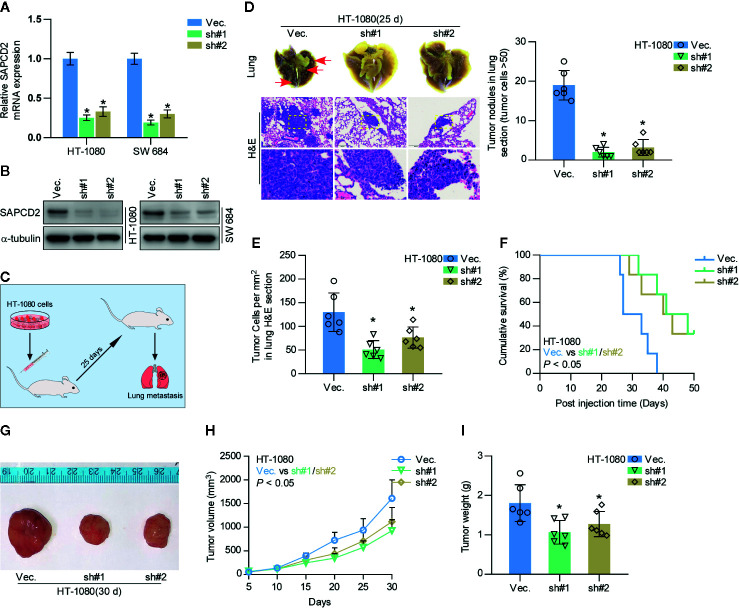
Silencing SAPCD2 inhibits lung metastasis of fibrosarcoma cells *in vivo*. **(A, B)** Real-time PCR and Western blotting analysis of SAPCD2 expression in the indicated fibrosarcoma cells. GAPDH was used as endogenous controls in RT-PCR and α-Tubulin was detected as a loading control in the Western blot. Each bar represents the mean values ± SD of three independent experiments. *P < 0.05. **(C)** Schematic model illustrating the time and route of HT-1080 cell (vec. or SAPCD2-sh#1/2 stable cell lines) administration in a mouse model of lung metastasis. **(D)** Representative images of the lung metastases formed and hematoxylin-eosin staining from the indicated cells in the mice. The numbers of lung tumor nests in each group was counted under a low power field and are presented as the median values ± quartile values (right panel). *P < 0.05. **(E)** The quantification of the number of fibrosarcoma cells (per mm^3^) in the indicated tumor tissues. Error bars represent the mean ± SD values. *P < 0.05. **(F)** Kaplan-Meier survival curves from the indicated mice groups. **(G)** Images of excised tumors from the mice at 30 days after injection with the indicated cells. **(H)** Tumor volumes were measured every 5 days. Each bar represents the median values ± quartile values. **(I)** Average weight of excised tumors from the indicated mice. Each bar represents the median values ± quartile values. *P < 0.05.

As a type of cancer with high metastatic potential, fibrosarcoma exhibits high avidity to metastasize to the lung (7–10). Hence, the effect of SAPCD2 on the local lung colonization of fibrosarcoma cells was further evaluated *in vivo*. We first knocked down the endogenous expression of SAPCD2 in HT-1080 and SW684 cells (**Figures 3A, B**), both of which express high levels of SAPCD2 (**Figure 1A**). A mouse model involving injection of HT-1080 cells *via* the tail vein was used to evaluate the effect of SAPCD2 on the lung metastasis of fibrosarcoma cells *in vivo*, and a schematic of the model is shown in **Figure 3C**. Mice were randomly divided into three groups (n = 6/group). Then, vector control HT-1080 cells and SAPCD2-silenced HT-1080 cells were injected into the tail veins of the mice. As shown in **Figures 3D–F**, compared with the vector control, silencing SAPCD2 not only reduced the formation of lung nodules and the number of cancer cells per mm^2^ but also extended the cumulative survival time of the mice. In subcutaneous xenografts, silencing SAPCD2 dramatically attenuated the primary growth and tumorigenic abilities of fibrosarcoma cells *in vivo*, as demonstrated by the reduced tumor volume and tumor weight in the mice inoculated with SAPCD2-silenced fibrosarcoma cells (**Figures 3G–I**). These findings demonstrated that silencing SAPCD2 inhibits the lung colonization and primary growth abilities of fibrosarcoma cells *in vivo*.

### Silencing SAPCD2 Inhibits Proliferation and Promotes Apoptosis

**Figure 4:**
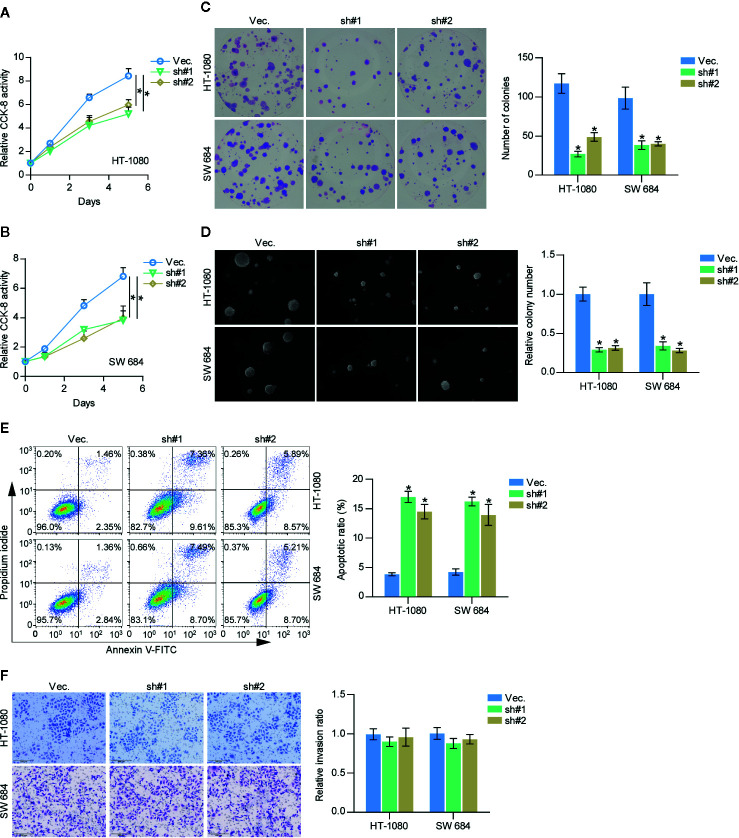
Downregulation of SAPCD2 retards the proliferation and anoikis resistance of fibrosarcoma cells *in vitro*. **(A, B)** The effect of silencing SAPCD2 on the proliferation rate in HT-1080 and SW684 cells by CCK-8 assay. Each bar represents the mean values ± SD of three independent experiments. *P < 0.05. **(C)** The effect of silencing SAPCD2 on the colony-forming ability in HT-1080 and SW684 cells by colony formation assay. **(D)** The effect of silencing SAPCD2 on the anchorage-independent growth ability in HT-1080 and SW684 cells by anchorage-independent growth assays. Each bar represents the mean values ± SD of three independent experiments. *P < 0.05. **(E)** The effects of SAPCD2 on apoptotic ratio in the indicated fibrosarcoma cells *via* annexin V-FITC/PI staining. Error bars represent the mean ± S.D. of three independent experiments. *P < 0.05. **(F)** The effect of SAPCD2 on the invasion ability in the indicated fibrosarcoma cells. Error bars represent the mean ± S.D. of three independent experiments.

A CCK-8 assay was first carried out to examine the effect of SAPCD2 downregulation on fibrosarcoma cells. The results showed that silencing SAPCD2 reduced the cell proliferation ability of HT-1080 and SW684 cells (**Figures 4A, B**). Moreover, silencing SAPCD2 decreased the colony-formation capability of HT-1080 and SW684 cells, as determined by colony formation assay (**Figure 4C**). An anchorage-independent growth assay was further carried out, and the results showed that silencing SAPCD2 inhibited the anchorage-independent growth ability of HT-1080 and SW684 cells (**Figure 4D**). Conversely, silencing SAPCD2 enhanced the apoptotic ratio of HT-1080 and SW684 cells (**Figure 4E**). In addition, silencing SAPCD2 had no significant effect on invasion ability of HT-1080 and SW684 cells (**Figure 4F**). Therefore, our results revealed that silencing SAPCD2 inhibits the proliferation and promotes the apoptosis of fibrosarcoma cells *in vitro*.

### Silencing SAPCD2 Activates the Hippo Signaling Pathway

**Figure 5:**
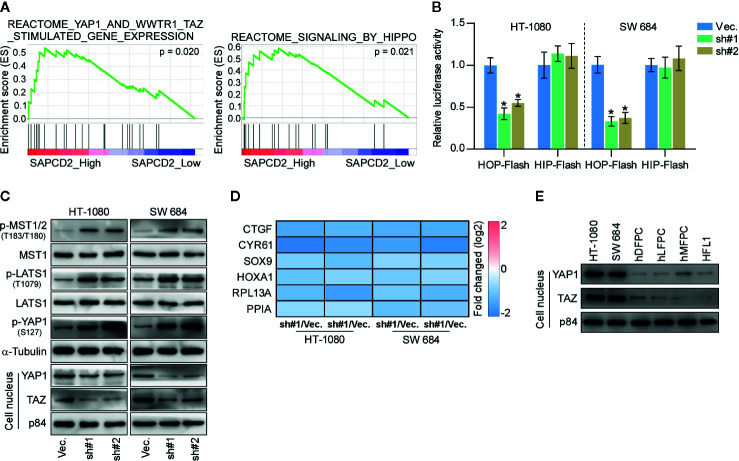
SAPCD2 inactivates Hippo signaling pathway. **(A)** GSEA analysis showed that SAPCD2 expression level was associated with the Hippo signaling. **(B)** TEAD transcriptional activity was assessed by HOP-Flash luciferase reporter in the indicated cells. Error bars represent the mean ± S.D. of three independent experiments. *P < 0.05. **(C)** Western blot analysis of total p-MST1/2, MST1, p-LATS1, LATS1, p-YAP1, YAP1 expression and nuclear YAP, TAZ expression in the indicated cells. α-Tubulin was detected as a loading control and nuclear protein p84 was used as the nuclear protein marker. **(D)** Real-time PCR analysis of CTGF, CYR61, SOX9, HOXA1, RPL13A, and PPIA in the indicated cells. Error bars represent the mean ± S.D. of three independent experiments. *P < 0.05. **(E)** Western blotting analysis of SAPCD2 expression in 4 normal fibroblast cell lines, including human dermal fibroblast primary cell (hDFPC), human lung fibroblasts primary cell (hLFPC), human mammary fibroblasts primary cell (hMFPC), Human embryonic lung fibroblast (HFL1), and 2 fibrosarcoma cell lines HT-1080 and SW684. α-Tubulin was detected as a loading control.

To further elucidate the underlying mechanism by which SAPCD2 regulates the lung colonization and cell proliferation of fibrosarcoma cells, GSEA was performed. As shown in **Figure 5A**, the expression of SAPCD2 was highly correlated with the signatures of Hippo signaling, supporting the notion that Hippo signaling may mediate the inhibitory effect of silencing SAPCD2 on the lung colonization and cell proliferation of fibrosarcoma cells. Luciferase reporter analysis showed that silencing SAPCD2 reduced the luciferase reporter activity of HOP-Flash, but not that of HIP-Flash, suggesting that silencing SAPCD2 inhibited TEAD-dependent luciferase activity in fibrosarcoma cells (**Figure 5B**). Western blotting analysis revealed that SAPCD2 downregulation increased the expression levels of phosphorylated MST1/2 (p-MST1/2), phosphorylated LATS1 (p-LATS1), and phosphorylated YAP1 (p-YAP1), and decreased the nuclear translocation of YAP and TAZ, which are downstream effectors of Hippo signaling; however, SAPCD2 downregulation had no effect on the total levels of MST1 and LATS1 in fibrosarcoma cells (**Figure 5C**). Real-time PCR analysis showed that silencing SAPCD2 decreased expression levels of multiple downstream genes of the Hippo pathway, including CTGF, CYR61, SOX9, HOXA1, RPL13A, and PPIA in fibrosarcoma cells (**Figure 5D**). Importantly, western blot analysis further revealed that the nuclear expression levels of YAP1 and TAZ were robustly upregulated in HT-1080 and SW 684 cells compared with four noncancerous fibroblast cell lines, including hDFPC, hLFPC, hMFPC, and HFL1 (**Figure 5E**). Taken together, these findings indicated that silencing SAPCD2 activates the Hippo signaling pathway in fibrosarcoma cells.

### Activation of the Hippo Signaling Pathway Mediates the Inhibitory Role of SAPCD2 Downregulation in the Proliferation of Fibrosarcoma Cells

**Figure 6:**
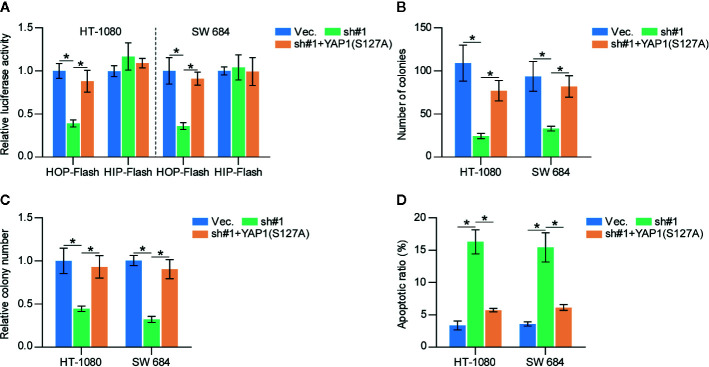
SAPCD2 promotes proliferation and anoikis resistance *via* inactivates Hippo signaling pathway in fibrosarcoma cells. **(A)** YAP1-S127A reversed the TEAD transcriptional activity repressed by downregulation of SAPCD2 assessed by HOP-Flash luciferase reporter. Each bar represents the mean values ± SD of three independent experiments. *P < 0.05. **(B, C)** YAP1-S127A reversed the inhibitory effects of SAPCD2 downregulation on colony-forming ability **(B)** and anchorage-independent growth capability **(C)** in fibrosarcoma cells. Each bar represents the mean values ± SD of three independent experiments. *P < 0.05. **(D)** YAP1-S127A reversed the pro-apoptotic role of SAPCD2 downregulation on in fibrosarcoma cells. Each bar represents the mean values ± SD of three independent experiments. *P < 0.05.

To determine the functional role of Hippo signaling in SAPCD2 downregulation-mediated inhibition of fibrosarcoma cell proliferation, constitutively active YAP1, YAP1-S127A (33), was added in SAPCD2-silenced HT-1080 and SW684 cells. First, YAP1-S127A dramatically increased the luciferase reporter activity of HOP-Flash in SAPCD2-silenced HT-1080 and SW684 cells (**Figure 6A**). Importantly, YAP1-S127A significantly reversed the inhibitory effect of SAPCD2 downregulation on the colony formation and anchorage-independent growth capabilities of HT-1080 and SW684 cells (**Figures 6B, C**). In contrast, YAP1-S127A markedly reduced the apoptotic ratio of SAPCD2-silenced HT-1080 and SW684 cells (**Figure 6D**). Collectively, these results indicated that silencing SAPCD2 inhibits proliferation and promotes apoptosis by activating the Hippo signaling pathway in fibrosarcoma cells.

## Discussion

The primary findings of the current study shed light on the critical role of SAPCD2 in the lung metastasis of fibrosarcoma. In the current study, our results demonstrated that overexpression of SAPCD2 was observed in fibrosarcoma tissues, and was associated with early progression and metastasis, and poor prognosis in fibrosarcoma patients. Silencing SAPCD2 inhibited the proliferation and increased the apoptosis of fibrosarcoma cells *in vitro*, and repressed the lung metastasis of fibrosarcoma cells *in vivo*. Mechanistic investigation further demonstrated that silencing SAPCD2 inhibited the proliferation and lung metastasis of fibrosarcoma cells by activating the Hippo signaling pathway. Therefore, our results determine an oncogenic role of SAPCD2 in the lung metastasis of fibrosarcoma.

Since its discovery, SAPCD2 has attracted great interest regarding its role in the context of cancer studies. SAPCD2 has been reported to be upregulated in various kinds of tumors, including breast cancer (34), gastric cancer (11), hepatocellular carcinoma (17), colorectal carcinoma (14), glioblastoma (19), nasopharyngeal carcinoma (15), lung adenocarcinoma (16), melanoma (18), and renal cell cancer (20). SAPCD2 further promotes progression and metastasis in different human types of cancer by activating the Wnt/β-catenin, JAK/STAT, TGF-β, MAPK, and NF-κB signaling pathways, all of which have been reported to play crucial roles in cancer progression and metastasis (35–40). However, the clinical significance and biological function of SAPCD2 in fibrosarcoma remain poorly known. In this study, our results showed that SAPCD2 is dramatically upregulated in clinical fibrosarcoma tissues, and this upregulation is significantly correlated with early progression and metastasis, and poor overall prognosis in fibrosarcoma patients. Loss-of-function assays showed that silencing SAPCD2 not only attenuated the proliferation and promoted the apoptosis of fibrosarcoma cells *in vitro*, but also inhibited the lung metastasis of fibrosarcoma cells *in vivo*. Mechanistically, our results further demonstrated that silencing SAPCD2 inhibited cell proliferation by the activating Hippo signaling pathway. Therefore, our findings in the current study reveal a novel mechanism by which SAPCD2 promotes the lung metastasis of fibrosarcoma, and identify a critical role of SAPCD2 in the lung metastasis of fibrosarcoma.

Several lines of evidence have reported that SAPCD2 may serve as a valuable prognostic marker in different types of cancer. A recent study by Jia and colleagues have reported that the PXN-AS1-L-induced increased expression of SAPCD2 by disrupting the binding of the microRNAs-mediated silencing complex to SAPCD2 predicted a worse prognosis in nasopharyngeal carcinoma patients (15). Moreover, SAPCD2 expression was reported to be significantly upregulated in breast cancer tissues, and its expression was further increased with advanced tumor stage. Importantly, breast cancer patients with high SAPCD2 expression had poorer overall survival rates than those with low SAPCD2 expression (34). However, the clinical significance of SAPCD2 in the progression and metastasis of fibrosarcoma is largely unknown. In the current study, our results showed that SAPCD2 expression was elevated in clinical fibrosarcoma tissues and was significantly correlated with early progression, distant metastasis, and poor overall survival in fibrosarcoma patients. Importantly, our results further demonstrated that silencing SAPCD2 inhibited the lung metastasis of fibrosarcoma cells in lung colonization models *in vivo*. Taken together, our findings suggest that SAPCD2 may have favorable prospects as a potential prognostic marker in fibrosarcoma. However, larger sample sizes in prospective studies are warranted in order to draw a more solid conclusion

As a tumor-suppressive pathway, inactivation of Hippo signaling has been extensively reported to be involved in the progression and metastasis of numerous types of cancer (41, 42), including fibrosarcoma (43–45). On the one hand, core components of the Hippo pathway MST1/2 and LATS1/2 were downregulated, and this downregulation was involved in the inactivation of Hippo signaling (46, 47); on the other hand, upregulation of the Hippo pathway transcription coactivators YAP and TAZ further exacerbated inactivation of the Hippo signaling, promoting the development and progression of cancers (48). To the best of our knowledge, in this study, we demonstrated for the first time that, silencing SAPCD2 activated the Hippo signaling in fibrosarcoma cells, as indicated by the reduced luciferase reporter activity of HOP-Flash, decreased nuclear expression of YAP and TAZ, decreased expression levels of multiple downstream genes of the Hippo pathway, and increased expression levels of p-MST1 and p-LATS1 in fibrosarcoma cells. Importantly, constitutively active YAP1, YAP1-S127A (33), significantly reversed the inhibitory effect of SAPCD2 downregulation on the colony formation and anchorage-independent growth capabilities of HT-1080 and SW684 cells, and markedly reduced the apoptotic ratio of SAPCD2-silenced HT-1080 and SW684 cells. Notably, a study by Zhang et al. has reported that inactivation of Hippo signaling mediated the role of SAPCD2 overexpression in promoting the viability, and invasion ability of breast cancer cells (34). Collectively, our findings in combination with existing reports indicate that the activation of the Hippo signaling pathway plays an important role in mediating the functional role of SAPCD2 in tumor growth and metastasis, at least in the context of of fibrosarcoma and breast cancer. However, the specific mechanism by which SAPCD2 inactivates Hippo signaling, which is a major shortcoming of the current study deserving further clarification in the future work.

In summary, our findings demonstrated that SAPCD2 functions as an oncogenic regulator to promote the proliferation and lung metastasis of fibrosarcoma cells by inactivating the Hippo signaling pathway. A better understanding of the underlying mechanism and the functional role of SAPCD2 in the pathogenesis of lung metastatic fibrosarcoma will facilitate the development of an anti-metastatic therapeutic strategy for the treatment of fibrosarcoma.

## Data Availability Statement

The original contributions presented in the study are included in the article/supplementary materials. Further inquiries can be directed to the corresponding authors.

## Ethics Statement

The studies involving human participants were reviewed and approved by Clinical ethics committee of Sun Yat-sen University. The patients/participants provided their written informed consent to participate in this study. The animal study was reviewed and approved by Animal ethics committee of Sun Yat-sen University.

## Author Contributions

WF and JL conceived the project and drafted the manuscript. BZ and Mingjun Bai conducted the experiments and contributed to the analysis of data. YW, LN, and WY analyzed the informatics data. MX and HW performed IHC and the analysis of data. JY edited and revised the manuscript. All authors contributed to the article and approved the submitted version.

## Funding

This study was granted by the National Natural Science Foundation of China (grant number 81701799) and Natural Science Foundation of Guangdong Province (grant number 2017A030310196).

## Conflict of Interest

The authors declare that the research was conducted in the absence of any commercial or financial relationships that could be construed as a potential conflict of interest.
